# Announcing *Molecular Biomedicine*

**DOI:** 10.1186/s43556-020-00003-2

**Published:** 2020-08-06

**Authors:** Arnold J. M. Driessen, Yu-quan Wei

**Affiliations:** 1 Faculty of Science and Engineering, University of Groningen, Groningen, Netherlands; 2 State Key Laboratory of Biotherapy and Clinical Cancer Center, West China Hospital, Sichuan University, Chengdu, China

*Molecular Biomedicine* is a peer-reviewed and open-access journal launched by Springer Nature in 2020, publishing the pioneer works in molecular medicine. We appreciate for the sincere support of many talented scientists and academic institutions worldwide that inspired us to start this journal.

Molecular medicine has a wide academic scope across basic, translational and clinical medicine, as well as physics, chemistry and biology from a molecular point of view to manage human diseases. The landmark event in the origin of molecular medicine was the achievement in 1949 published by Linus Carl Pauling, the two-times Nobel Prize laureate, who pointed out that sick cell anemia was a molecular disease caused by the chemical modification of a single well-identified protein. In 1990s, the launch of Human Genome Project guided molecular medicine into a new era, leading to the progress of epigenetics, genomics, transcriptomics, proteomics and metabolomics, as well as the invention of novel biotechnologies such as CRISPR/Cas9, single-cell sequencing and machine learning. Driven by these advances, many novel pathogenic factors including DNA methylations in cell development, non-coding RNAs in cancer, and protein post-translational modifications in neurodegenerative disease, were identified, promoting the rational understanding of the molecular mechanism in pathogenesis. Under the efforts of translational medicine research in recent years, many cutting-edge theories and technologies have been transformed into products that improve human health, such as cobas EGFR Mutation Test (Roche, Basel, Swiss) for the liquid biopsy of non-small cell lung cancer, Keytruda (Merck, Kenilworth, USA) for cancer therapy, and Praluent (Sanofi, Paris, France) for hypercholesterolemia treatment. The emergence of these products in turn triggers the progress of molecular medicine.

Indeed, molecular medicine is a rapidly developing realm of research. The number of papers published each year in this field has dramatically increased over the past years. Many governments and foundations around the world are increasingly providing financial support to the research in molecular biomedicine, aiming to improve the diagnosis, prevention and treatment of human diseases. Undoubtedly, numerous important discoveries will be accomplished in the near future, but the limited number of existed high-impact journals in this field cannot ensure the timely publication of relevant works that will emerge at an increasing rate. On this basis, *Molecular Biomedicine* is established to meet the current and future demand.

The goal of *Molecular Biomedicine* is to publish the significant findings across the full spectrum of medicine, such as clinical medicine, genetics, bioinformatics, molecular biology, cell biology, pharmacology, medicinal chemistry and pharmacy. This journal covers major human diseases including cancer, cardiovascular diseases, diabetes, neurodegenerative diseases, bacterial and virus infection, and chronic bronchitis, particularly emphasizing on the molecular process to disclose innate cause of diseases and suggest new techniques for diagnosis and therapy. The scope of *Molecular Biomedicine* includes but is not limited to epigenetics, genomics, proteomics, gene editing, RNA biology, signal transduction, structural biology, early diagnosis and biomarkers of disease, gene therapy, cell therapy and immunotherapy, targeted therapy by small molecules, molecular imaging, artificial intelligence in medicine and regenerative medicine.

We aim to establish a leading platform for medical research and offer the latest advances to meet the needs of academic readers. A range of content types such as original research articles, reviews, letters-to-editor, and research highlights related to the original discoveries or critical thinking in biomedicine are interested by *Molecular Biomedicine*, suggesting a new opportunity to share information across the specialists and the public.

*Molecular Biomedicine* has a globally renowned Editorial Board members composing of high-impact scientists from all over the world. We welcome researchers to submit valuable works to *Molecular Biomedicine* and promise to give a fair and prompt first decision within 3 weeks after submission. Our editorial staffs will also provide high-quality support to ensure your article to publish online within 2 weeks after formal acceptance.

In summary, with advances in new technologies including high-throughput DNA/RNA sequencing, gene editing, artificial intelligence and nano-biomaterials, we have entered an unprecedented era, in which the molecular process of life is increasingly understood, and precise biomarkers and targeted therapies are timely emerged. *Molecular Biomedicine* gives a forum for students, scientists, and clinicians interested in biomedicine to share their discoveries and viewpoints. We would complete our peer-review process as quick as possible, publish high-quality papers, and would like to invite you to submit your manuscripts to *Molecular Biomedicine*.

